# Exploring Drivers of Liking of Low-Phenylalanine Products in Subjects with Phenyilketonuria Using Check-All-That-Apply Method

**DOI:** 10.3390/nu10091179

**Published:** 2018-08-28

**Authors:** Cristina Proserpio, Ella Pagliarini, Juri Zuvadelli, Sabrina Paci, Alice Re Dionigi, Giuseppe Banderali, Camilla Cattaneo, Elvira Verduci

**Affiliations:** 1 Department of Food, Environmental and Nutritional Sciences (DeFENS), University of Milan, 20133 Milan, Italy; ella.pagliarini@unimi.it (E.P.); camilla.cattaneo@unimi.it (C.C.); 2 Department of Paediatrics, San Paolo Hospital, Department of Health Sciences, University of Milan, 20142 Milan, Italy; ju.zuva@gmail.com (J.Z.); sabrina.paci@asst-santipaolocarlo.it (S.P.); alice.redionigi@yahoo.it (A.R.D.); giuseppe.banderali@unimi.it (G.B.); elvira.verduci@unimi.it (E.V.)

**Keywords:** acceptability, food development, sensory attributes, CATA, dietotherapy, aromas

## Abstract

The aim of the present study was to apply the Check-all-that-apply (CATA) method in an ambulatory context involving subjects with phenylketonuria (PKU) to obtain a sensory description and to find the drivers of liking of low-phenylalanine products (Glycomacropeptide vs. L-amino acids formulas). 86 subjects with PKU (age range: 8–55 years) evaluated 8 samples: 4 L-amino acid formulas and 4 Glycomacropeptide (GMP) formulas, flavored with neutral, chocolate, strawberry and tomato aromas. Participants were asked to indicate which sensory attributes characterized each formulations and to score the overall liking. Significant differences were found regarding liking scores (F = 65.29; *p* < 0.001). GMP samples flavored with chocolate and strawberry, described as sweets, with a mild and natural taste and odor, were the most appreciated. Overall, GMP formulas obtained higher liking scores compared to L-amino acid formulas. Tomato flavored samples, described as bitter, salty, with artificial color, with strong taste and odor, obtained the lowest scores. In conclusion, CATA questionnaire seems to be a suitable method also in ambulatory context since this approach suggested that different foods and beverages with GMP could be developed to improve dietary treatment compliance of subjects with PKU from school age onwards.

## 1. Introduction

Phenylketonuria (PKU; Online Mendelian Inheritance in Man, OMIM 261,600) is an autosomal recessive disorder of phenylalanine (Phe) metabolism [1], primarily due to mutations in Phe hydroxylase (PAH) gene, which facilitates conversion of the essential amino acid Phe to tyrosine. Loss of PAH activity results in increased Phe concentrations in the blood (hyperphenylalaninaemia, HPA) and therefore in toxic concentrations in the brain. The main goal of treatment for PKU is to maintain the blood Phe within safe limits to prevent mental retardation and ensure normal growth and life with good health through adulthood. The dietary treatment usually begins immediately after confirmation of PKU diagnosis in newborns and should be continued throughout their lifetime in patients with untreated phenylalanine levels more than 600 μmol/L. Compliance with treatment is adequate in infancy and childhood, however difficulties in maintaining a PKU diet in adolescent and adulthood are reported [2]. Patients with PKU have to avoid foods rich in protein (e.g., meat, fish and dairy products), thus the diet consists mainly of low-protein natural foods (vegetables, fruits) and special low protein products, such as bread and pasta with a protein content <1 g/100 g. The required amount of daily protein is obtained from Phe-free protein substitutes providing essential amino acids in suitable proportions [3]. The number of protein substitutes (mixtures of amino acids that are free from or low in Phe) available for PKU patients is increasing constantly over time [4,5].

Even if improvements in the palatability, presentation, convenience and nutritional composition of substitutes have helped to improve long-term compliance with PKU diet, the acceptability of these substitutes remains a critical point, thus further improvement in this area is needed.

Casein Glycomacropeptide (GMP) is a protein derived from cheese whey that is rich in specific essential amino acids but it is the only known natural protein that in its purified form is free of tyrosine, tryptophan and phenylalanine. Therefore, GMP could provide an alternative protein source for PKU individuals when manufactured to sufficient purity to ensure the absence of Phe [6].

Studies in literature that consider the sensory analysis of low protein recipes for PKU dietotherapy are scarce. Moreover, these researches generally focused only on the overall acceptability of GMP products applying unsuitable hedonic methods [7,8]. Indeed, in the mentioned studies PKU subjects’ sample size was not appropriate to perform a hedonic evaluation, since the subjects involved were less than the required number. The lack of empirical studies regarding the patients’ satisfaction of the low-phenylalanine products and the requirement of new approaches for dietary management of PKU, reinforce the need to evaluate methods for studying PKU patients’ perception about the sensory characteristics of low protein products. In this context, the Check-all-that-apply (CATA) questionnaire could be an alternative approach for this purpose, since it has been proposed as a valid and rapid method for obtain a descriptive profile from consumers [9]. Indeed, with this exploratory approach a larger number of attributes, compared to other sensory evaluation such as the Just-about-right scale performed on a set of well-known products focusing on few attributes, can be evaluated to identify those with greatest impact on hedonic product performance [10].

To our knowledge, there are no studies using valid method to evaluate and obtain a sensory characterization of low-phenylalanine products, especially in an ambulatory context.

The first aim of this study was to investigate the liking of low-phenylalanine products comparing L-amino acid formulas and GMP formulas. The second aim was to obtain a sensory description of the desirable and undesirable sensory properties of these products. For this purpose, the CATA questionnaire was used to identify PKU subjects’ drivers of liking.

## 2. Materials and Methods

### 2.1. Participants

A total of 86 subjects with PKU (range age: 8–55 years), 45 males and 41 females, were recruited among patients referred to the Department of Pediatrics, San Paolo Hospital (Milan, Italy).

All participants had a biochemical diagnosis confirmed by genetic investigation and 6 participants were late diagnosed while for all others neonatal screening was made. The characteristics of the population are summarized in Table 1.

All participants were following a low-phenylalanine diet (84% of the participants were following a diet mainly based on L-amino acid formulas, 7% of the participants were not using a medical integration, 8% of the participant were following a diet based on both L-amino acid formulas and GMP, only one participant was using GMP). PKU children were defined as compliant to the diet when the annual mean Phe levels, monitored monthly by the Guthrie test, was within the range 120–360 mmol/L in childhood (<12 years) and 120–600 mmol/L in adolescence and adult age (>12 years) [2].

The exclusion criteria were: pregnancy, food allergies to whey proteins, severe neurological and functional disorders. Every subject was asked for informed consent before making the assessments. The present study was performed according to the principles established by the Declaration of Helsinki after the protocol was approved by the Institutional Ethics Committee (protocol approval n°210).

### 2.2. Samples

Eight low protein recipes for PKU dietotherapy were used. The GMP formula (GMP_N; nutritional composition: 9 g carbohydrates of which 7 g sugars, 1.4 g fat and 5 g protein per 100 mL) was made using 100 mL of Glytactin RTD^TM^ (Cambrooke Therapeutics, MA, USA) whereas the L-amino acid formula (AA_N; nutritional composition: 43 g carbohydrates of which 3 g sugars, 14 g fat and 5 g protein per 100 g) was made mixing 16.5 g of powder high in L-amino acid (Xphe energy kid neutral, MetaX, Dietetic Metabolic Food: DMF, Limbiate, Monza Brianza, Italy) and water to reach a final volume of 100 mL.

The flavored versions were prepared by adding 2 g of flavoring powder to these neutral formulations. In particular, strawberry aroma (aroMaxx erdbeere, MetaX, DMF, Limbiate, Monza Brianza, Italy) or tomato and basil aroma (aroMaxx tomate-basilikum, DMF, Limbiate, Monza Brianza, Italy) were added to GMP-base formula (GMP_S and GMP_T; respectively), and chocolate aroma (aroMaxx schoko, MetaX, DMF, Limbiate, Monza Brianza, Italy) or tomato and basil aroma (aroMaxx tomate-basilikum, DMF, Limbiate, Monza Brianza, Italy) were added to L-amino acid formula and water (AA_C and AA_T; respectively). The GMP chocolate flavored sample (GMP_C) were prepared using 100 mL of Glytactin RTD^TM^ Chocolate (Cambrooke Therapeutics, Massachusetts, USA) and the L-amino acid strawberry flavored sample (AA_S) were prepared using 16.5 g of Xphe energy kid erdbeere (MetaX, DMF, Limbiate, Monza Brianza, Italy) and water. Each of these samples provides 5 g/100 mL protein equivalents.

A detailed composition of the samples is reported in Table 2.

The eight samples were presented as beverages to participants following a randomized and balanced order for each participant. Approximately 30 mL of each sample were presented to the participants monadically in plastic cups labelled with three-digit codes. Water was available for rinsing the palate.

### 2.3. Experimental Procedure

All the evaluations were performed in a quiet room and all the participants were tested at the same time (10:30–12:30). They were asked to refrain from consuming anything but water for 2 h before the test (hungry state). For each sample, subjects had to score their overall liking and to answer a check-all-that-apply (CATA) questionnaire.

### 2.4. Liking Assessment

Participants were asked to taste the products monadically and to express their liking scores. Children (aged 8–12 years) were asked to express their liking through a vertical 7-point facial hedonic scale, from “super good” (7) to “super bad” (1) [11], whereas subjects aged between 13 and 65 years rated their liking using a 10cm Visual Analogue Scale (VAS) anchored by the extremes ‘‘extremely disliked” (rated 0) and ‘‘extremely liked” (rated 10).

### 2.5. Check-All-That-Apply (CATA) Assessment

The CATA questionnaire consisted on a list of 27 sensory attributes including appearance, odor, taste, flavor and texture terms. Participants were asked to check from the list all the terms that they considered appropriate to describe each of the samples. The terms considered were the following: 10 for the appearance (light brown, dark brown, light yellow, dark yellow, light pink, dark pink, natural color, artificial color, brightness and opaque), 6 for the odor (natural odor, artificial odor, mild odor, strong odor, milk odor and vanilla odor), 8 for the taste/flavor (sweet, bitter, salty, sour, mild taste, strong taste, milk flavor and vanilla flavor) and 3 for the texture (thin, thick and floury). The position of attributes was randomized using the “to assessor” list order allocation scheme [12].

A separate group of 12 untrained PKU subjects aged 20–40 years’ old took part in a pilot test, wherein judges used a free listing questionnaire to establish the appropriate terms to describe the samples [13]. They were provided with the eight formulations and for each sample, they were asked to pay attention to the sensory characteristics and to write all terms for describing their color, appearance, odor, taste, flavor and texture. An open discussion followed the development of lexicon. Then the experimenters finalized the list of terms, selecting the most mentioned and the most common word in order to avoid synonymous [14].

### 2.6. Data Analysis

A mixed ANOVA was carried out on overall liking data considering ‘samples’ (GMP_N; GMP_S; GMP_C; GMP_T; AA_N; AA_S; AA_C and AA_T), ‘gender’ (women and men), ‘age’ (young: <21 years old; adults: ≥21 years old) and their two-way interaction (‘sample’ × ‘gender’; ‘sample’ × ‘age’) as fixed factors. Liking provided by children where adjusted by using a proportion in order to have results comparable to those provided by adults. In order to examine how the adherence to the dietotherapy affects the liking of the evaluated low-phenylalanine formulas a model was constructed with ‘adherence to diet’ (‘good adherence’ = Phe levels 120–360 mmol/L in children < 12 years, and 120–600 mmol/L in adolescence and adult age >12 years; ‘scarse adherence’ = Phe levels > 360 mmol/L in children < 12years, and >600 mmol/L in adolescence and adult age > 12 years) (van Spronsen et al. 2017) and ‘samples category’ (GMP and AA) and their two-way interaction as fixed factors. This analysis has also been performed considering only GMP samples which obtained the higher liking scores and the L-amino acid samples flavored with the same aroma. Thus, a model was constructed with ‘adherence to diet’ and ‘samples’ (GMP_S; GMP_C; AA_S; AA_C) and their two-way interaction as fixed factors. Participants were added as random factor in all the analyses. When a significant difference (*p* < 0.05) was found, least significant difference (LSD) *post hoc* test was used. These statistical analyses were performed using IBM SPSS Statistics for Windows, Version 24.0 (IBM Corp., Armonk, NY, USA).

For the CATA question, frequency of mention for each term was determined by counting the number of participants that used that term to describe each sample. Cochran’s *Q* test was carried out for each of the 27 terms to detect differences in participants’ perception of the evaluated samples. Correspondence analysis (CA) was performed to study the relationship between CATA questions and liking data. CA was performed on the frequency table containing responses to the CATA questions, considering the average liking scores by product as supplementary variable. These statistical analyses were performed using XLSTAT-Sensory^®^ software for Windows, Version 2015.6.01 (Addinsoft^™^, Paris, France). A *p*-value of <0.05 was considered significant.

## 3. Results

### 3.1. Liking Assessment

**Figure 1:**
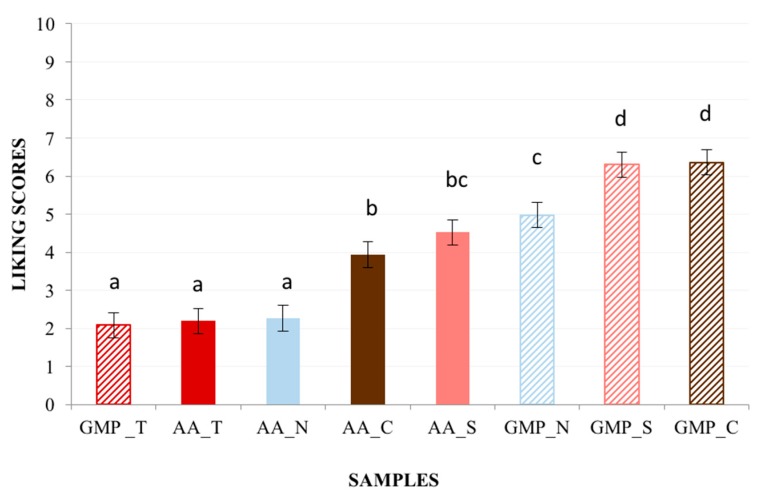
Liking score ± standard error mean (SEM) by samples. Different letters indicate significant differences according to *post hoc* test (Patients *n* = 86).

The mean liking scores by samples are provided in Figure 1. The main factor ‘samples’ was found to have a significant effect on liking (F_(7,581)_ = 65.29, *p* < 0.001). Overall, GMP formulas obtained higher liking scores (M = 4.84 ± 0.18) compared to the L-amino acid formulas (M = 3.06 ± 0.18). In particular, GMP_S (M = 6.27 ± 0.26) and GMP_C (M = 6.27 ± 0.26), which were comparable to each other, obtained the highest liking scores. GMP_T (M = 2.06 ± 0.26), AA_T (1.81 ± 0.26) and AA_N (M = 2.15 ± 0.26), which were comparable to each other, obtained the lowest hedonic ratings and were not acceptable since they were below the middle of the scale.

**Figure 2:**
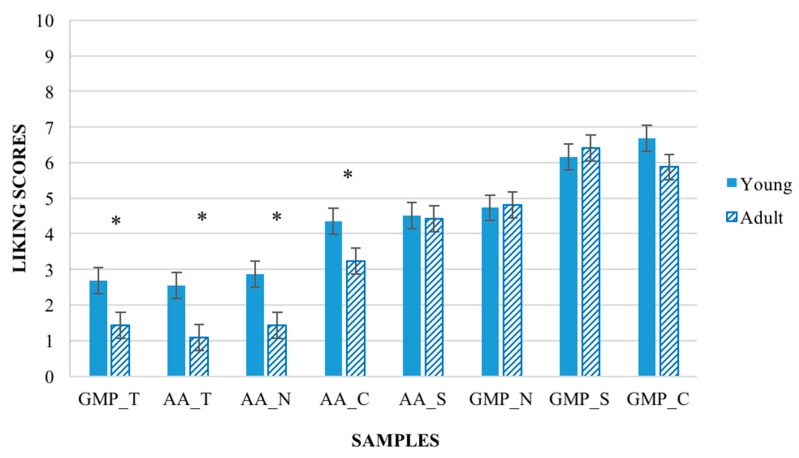
Liking score ± SEM by samples × age. Significant difference for * *p* < 0.05 (Young patients *n* = 43; Adult patients *n* = 43).

The main factor ‘age’ was found to have a significant effect on liking (F_(1,83)_ = 5.96, *p* = 0.02). Overall, the young participants provided higher liking scores (M = 4.31 ± 0.21) to the samples compared to the adult participants (M = 3.59 ± 0.21). Moreover, the interaction ‘samples’ × ‘age’ had a significant effect on liking scores (F_(7,581)_ = 2.45, *p* = 0.02). As shown in Figure 2, considering the less preferred samples (GMP_T, AA_T, AA_N, AA_C) young participants gave significant higher liking scores (*p* < 0.05) compared to adult participants.

The main factor ‘gender’ and the interactions ‘sample’ × ‘gender’ were not significant (F_(1,83)_ = 0.07, *p* = 0.80; F_(7,581)_ = 0.45, *p* = 0.87, respectively).

**Figure 3:**
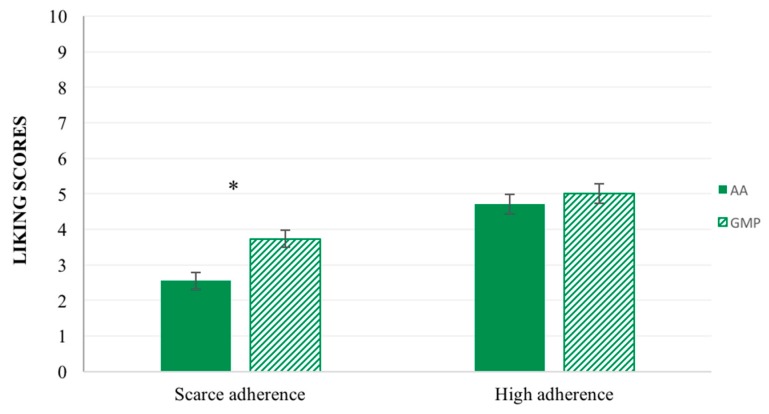
Liking score ± SEM by ‘adherence to diet’ × ‘samples category’. Significant difference for * *p* < 0.05 (Patients with scarce adherence *n* = 49, Patients with high adherence *n* = 37).

A significant effect of the main factor ‘adherence to diet’ on liking scores was found (F_(1,84)_ = 6.10, *p* = 0.02). Generally, participants with ‘scarce adherence’ to the dietotherapy gave significant lower liking scores (M = 3,63 ± 0.19) compared to participants with ‘good adherence’ (M = 4.37 ± 0.23). Moreover, the interaction ‘adherence to diet’ × ‘samples category’ had a significant effect on liking (F_(1,600)_ = 5.06, *p* = 0.02). In particular, as shown in Figure 3, participants characterized by ‘scarce adherence’ gave significant higher liking scores to the GMP formulas (M = 3.74 ± 0.27) compared to the L-amino acid formulas (M = 2.55 ± 0.24). 

**Figure 4:**
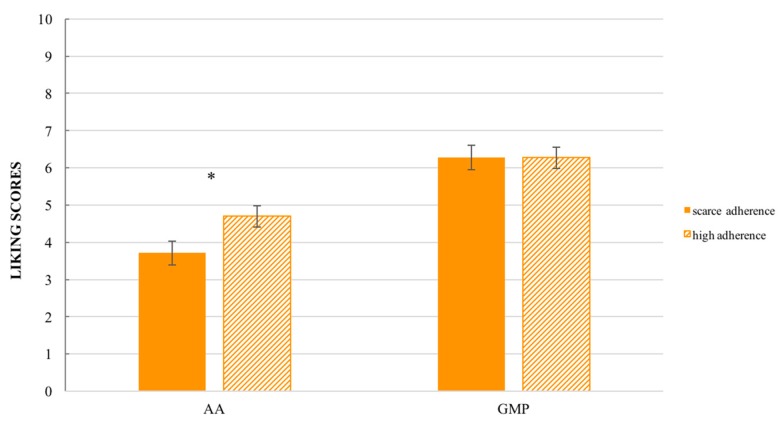
Liking score ± SEM by ‘adherence to diet’ × ‘samples’ (AA_C, AA_S, GMP_C, GMP_S). Significant difference for * *p* < 0.05. (Patients with scarce adherence *n* = 49, Patients with high adherence *n* = 37).

Considering only GMP samples which obtained the higher liking scores (GMP_C and GMP_S) and the L-amino acid samples flavored with the same aroma (AA_C and AA_S) and the ‘adherence to diet’ a significant ‘sample’ effect was found (F_(1,256)_ = 64.83, *p* < 0.001). As shown in Figure 4 patients characterized by a scarce adherence to diet gave comparable liking scores to GMP samples compared to patients with high adherence to the diet. Contrarily, patients with scarce adherence to diet gave significant (*p* < 0.05) lower liking scores to AA samples compared to subjects with high adherence to diet. The main factor ‘adherence to diet’ and the interaction ‘adherence to diet’ × ‘samples category’ were not significant (F_(1,84)_ = 1.99, *p* = 0.16; F_(1,256)_ = 3.75, *p* = 0.06, respectively).

### 3.2. CATA Assessment 

The frequency table of terms checked by patients to describe the eight samples is reported in Table 3.

As shown in Table 3 significant differences were found in the frequency for 25 out of 27 terms within the five categories considered, suggesting that participants perceived differences between samples in terms of their sensory characteristics. The sensory attributes that were not useful in order to discriminate samples were ‘artificial color’ and ‘natural odor’. Indeed, looking at the frequency of mention of these attributes these terms were used quite homogeneously and were checked by less half of the respondents, indicating that the participants’ consensus was low.

### 3.3. Relating Sensory Profiling (CATA) with Liking

The purpose of this calculation was to establish which sensory attributes are mainly related to the overall liking of the samples and to obtain a perceptual map of the products based on both liking and sensory profiling.

**Figure 5:**
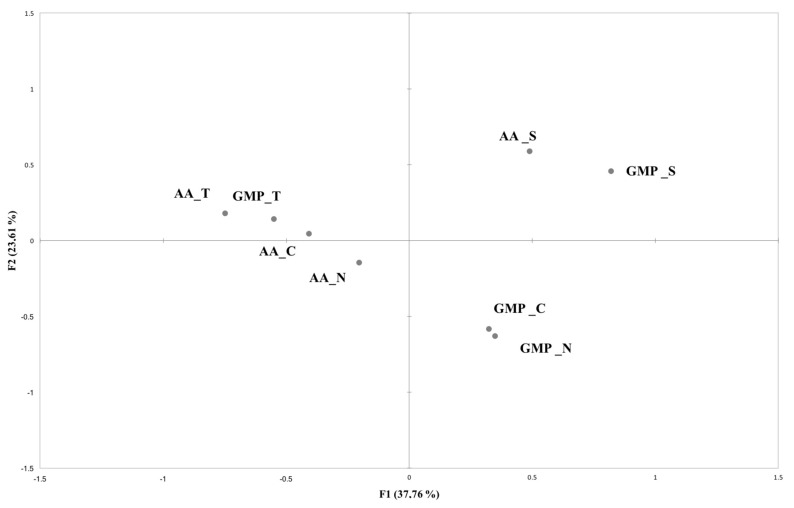
Products plot obtained from Check-all-that-apply (CATA).

The CA performed on the total frequency participants counts for each attributes resulted in two dimensions accounting for 60.43% of variance in the data. As inferred from the product plot (Figure 5), samples were discriminated according to their flavor, with samples with strawberry aroma (AA_S and GMP_S) in the upper right side of the map while the samples added with tomato aroma were positioned in the upper left side of the map (AA_T and GMP_T). Looking at the lower part of the map GMP formulas without aroma (GMP_N) and the chocolate one (GMP_C) are well distinguished from the L-amino acid formulas with the same aromas (AA_N and AA_C).

**Figure 6:**
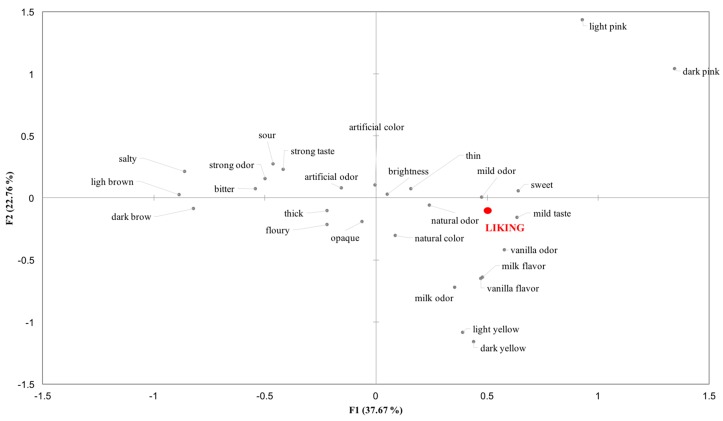
Attributes plot obtained from CATA total frequency counts and liking. Underline terms described samples that obtained higher liking scores while terms in italic describe mainly the disliked samples.

The relation between sensory terms and overall liking of the eight samples is reported in Figure 6.

Comparing Figure 5 and Figure 6, it is possible to see that participants liking was oriented toward GMP_C and GMP_S on the right side of the map, which were mainly associated with the sensory attributes ‘sweet taste’, ‘mild taste’ and ‘mild odor’, and ‘natural odor’. Liking was negative related to ‘bitter taste’, ‘strong taste’, ‘salty’, ‘strong odor’, ‘artificial odor’, ‘light brown’ and ‘artificial color’, which described the samples that obtained the lowest liking scores (GMP_T, AA_T and AA_N).

## 4. Discussion

The aim of the present study was to perform the Check-all-that-apply (CATA) method in an ambulatory context involving subjects with phenylketonuria (PKU) to obtain a sensory description and to find the drivers of liking of low-phenylalanine products (Glycomacropeptide vs. L-amino acids formulas).

We demonstrated a greater acceptability of GMP beverages compared to the amino acid formulas currently required as the primary source of protein in the PKU diet [15]. Moreover, GMP samples flavored with chocolate and strawberry, described as sweets, with a mild and natural taste and odor, were the most appreciated. Contrarily, tomato flavored samples, described as bitter, salty, with artificial color, with strong taste and odor, obtained the lowest liking scores.

Liking scores between women and men were comparable to each other while young subjects provided generally higher liking scores compared to the adults. Accordingly, it is well known that subjects become more critical in their food choices and preferences with increasing age [16], maybe due to a greater exposure to a wide range of food products.

Previous studies explored the overall acceptability of GMP products, but no one investigated which sensory properties characterized these products [7,8,17]. None of the mentioned studies measured acceptability with an appropriate method or with a representative sample size. Indeed, Lim and collaborators (2007) showed that a GMP chocolate beverage was significantly more acceptable compared to the same flavored amino acid beverage. However, PKU subjects’ sample size was not appropriate to perform a hedonic evaluation, since the subjects involved were less than the required number. Similarly, van Calcar and colleagues [8] concluded that, in a group of only 10 subjects involved in an 8-day inpatient metabolic study, the GMP products were better tasting compared with usual amino acid formulas, both consumed during the treatments. Again, this assumption was not supported by a proper hedonic evaluation, since, besides the small group of subjects recruited, any quantitative sensory evaluation was performed. Recently, Ney and colleagues (2016) in a randomized crossover trial with 30 early-treated phenylketonuria subjects found, using a questionnaire, that GMP samples were generally more acceptable than AA formulas. However, a sensory evaluation was not performed to confirm these results. From a methodological point of view, this is the first study that included an adequate sample of subjects with PKU and fulfilled the requirements to perform a sensory description of products using the CATA approach [9]. In line with the literature data, an appropriate sample size was recruited [18] and a suitable list of CATA questions was used. Indeed, in order to consider consumer heterogeneity and avoiding a dilution effect of the responses, it has been reported that a minimum of 10 to a maximum of 40 terms should be comprised in the list [14,19].

Subjects’ CATA counts were significantly different for the evaluated samples suggesting that this technique was able to detect differences in subjects’ perception of low protein products. Thus, CATA questionnaire may represent an alternative and rapid method for the description of products’ sensory characteristics, when it is difficult to apply traditional sensory descriptive analyses (e.g., sensory profile). This new approach has been already successfully applied to evaluate sensory perception of a food product in particular context or with specific group of subjects [20,21]. Indeed, De Pelsmaeker and collaborators [20] used this method with 8- to 13-year-old children to obtain emotional profiles of food, and Laureati and collaborators [21] tested CATA questions in a natural context (e.g., school) as an alternative approach to descriptive methods in food product development with a young panel.

Present results support the feasibility of GMP in making a great selection of palatable foods and beverages to improve the taste, variety and compliance of the PKU diet, with a positive impact on it. In addition, it has been suggested the ability of GMP to promote a greater satiety when compared to amino acid-based formulas and to suppress plasma ghrelin levels in individuals with PKU [8,22].

Regarding variety, one the biggest obstacle to following the PKU diet is that the amino acid mixtures are usually available and consumed as a liquid formula [23]. On the contrary, GMP is well suited for use not only in beverages but also in semi-solid foods [24]. Indeed, various low-phenylalanine products such as beverages, pudding and crackers has been developed [15], due to its functional properties including good heat stability and solubility in acid [25].

Moreover, the main goal in PKU treatments is to facilitate long-term dietary compliance and to ultimately improve quality of life and metabolic control for individuals with PKU. Indeed, data in literature shown that compliance with treatment seems to be adequate in infancy and childhood [26] but scarce adherence and difficulties in maintaining PKU diet have been reported in patients above 16 years of age, in whom Phe values were within or below guideline goals [27]. Even if, evidences suggested that GMP is more acceptable than the traditional amino acid mixtures it is not an easy task to change patients’ food habits trough these new formulations. Indeed, it is well known that food habits are difficult to be changed and PKU patients are used to drink amino acid mixtures since infancy. PKU is an extreme example of a well-established eating pattern. This suggests that subjects with PKU who are compliant with consuming AA formulas are imprinted with a preference for the taste and emotional components associated with lifelong consumption these formulas [17]. Indeed, Ney and colleagues (2016) showed that patients used to consumed the amino acid mixtures stated that GMP tasted better but that they still craved AA formulas. Moreover, even if it has been shown that in PKU mouse model the ingestion of GMP decreased Phe concentrations in blood and increased the brain tissue, researches are ongoing to evaluate the long-term safety and efficacy of GMP in the nutritional management of PKU [2,28].

The present data suggested that subjects with a scarce adherence to diet preferred generally the GMP formulas compared to the commonly amino acid mixtures, supporting the hypothesis that the implementation of the diet with more appreciated products could maybe enhance their dietary compliance, improving also subjects’ health status, especially in adults.

## 5. Conclusions

In conclusion, the sensory approach through the CATA method could be useful to understand how implements dietotherapy of subjects with PKU, considering their satisfaction as one of the main aspects during the product development. As future perspective, it could be useful for industries to develop new GMP products taking into account the information related to the sensory perception, specially to taste and odor attributes, in order to satisfying at the same time both nutritional and sensory aspects. Thus, the low-Phe formulas should have mild odor and taste, they should be sweet and with a more natural odor compared to the traditional mixtures.

## Figures and Tables

**Table 1:** Characteristics of participants.

Variable	PKU ^a^ 8–12 years (*n* = 18)		PKU > 13 years (*n* = 68)	
	Mean (SD ^b^)	Median (25th–75th Centile)	Mean (SD)	Median (25th–75th Centile)
Metabolic Control				
Phe (µmol/L)	268.5 (72.4)	272.6 (204.2–277.4)	569.0 (325.4)	471.9 (338.2–738.5)
Anthropometry ^c^	Childhood and adolescence (*n* = 30)		Adult (*n* = 56)	
	Mean (SD)	Median (25th–75th centile)	Mean (SD)	Median (25th–75th centile)
BMI ^d^ (kg/m^2^ )			22.9 (4.6)	21.8 (19.7–25.6)
BMI Z-score	0.53 (1.0)	0.44 (−0.32–1.29)		
Underweight (%)	0		10.7	
Normal-weight (%)	72.4		67.8	
Overweight (%)	17.2		12.5	
Obese (%)	10.3		8.9	

^a^ PKU: phenylketonuria; ^b^ SD: standard deviation; ^c^ according to WHO (http://www.who.int/en/); ^d^ BMI: body mass index.

**Table 2:** Compositions of the eight samples.

Samples	Composition
**GMP ^a^ formulas**	
GMP_N	50 mL Glytactin 10 RTD ^b^ neutral + 50 mL Glytactin 15 RTD neutral
GMP_C	50 mL Glytactin 10 RTD chocolate + 50 mL Glytactin 15 RTD chocolate
GMP_S	50 mL Glytactin 10 RTD neutral + 50 mL Glytactin 15 RTD neutral + 2 g strawberry aroma
GMP_T	50 mL Glytactin 10 RTD neutral + 50 mL Glytactin 15 RTD neutral + 2 g tomato and basil aroma
**L-amino acid formulas**	
AA_N	16.5 g Xphe energy kid neutral + water
AA_C	16.5 g Xphe energy kid neutral + 2 g chocolate aroma + water
AA_S	16.5 g Xphe energy kid erdbeere + water
AA_T	16.5 g Xphe energy kid neutral + 2 g tomato and basil aroma + water

^a^ GMP: glycomacropeptide; ^b^ RTD: ready to drink.

**Table 3:** Frequency mention of sensory attributes associated with each samples.

Sensory Modality	Sensory Attributes	Frequency of Mention							
		Samples							
		AA_C	AA_S	AA_N	AA_T	GMP_C	GMP_S	GMP_N	GMP_T
Appearance	Artificial color ^n.s.^	19	26	17	19	18	18	19	25
	natural color ***	11	10	27	10	20	10	23	8
	light yellow ***	0	0	14	1	22	0	34	3
	dark yellow ***	0	0	1	0	21	0	4	4
	brightness *	8	20	14	15	13	10	19	14
	light brown ***	5	0	0	62	25	0	3	61
	dark brown ***	81	0	0	14	3	0	0	7
	opaque ***	28	9	14	15	22	14	20	17
	light pink ***	0	79	0	0	0	23	0	4
	dark pink ***	0	6	0	0	0	60	0	2
Odor	artificial odor *	32	28	36	33	19	22	23	31
	mild odor ***	20	34	12	5	27	29	31	7
	milk odor ***	4	3	16	1	25	7	30	7
	vanilla odor ***	2	9	4	2	17	9	20	3
	strong odor ***	22	15	21	56	16	17	12	53
	natural odor ^n.s.^	13	11	10	8	16	16	12	5
Taste	sweet ***	27	43	9	5	54	70	34	10
	sour ***	13	19	29	24	4	6	4	31
	salty ***	11	9	23	55	4	1	5	49
	bitter ***	32	14	37	32	1	3	13	20
	mild taste ***	17	23	5	3	42	46	38	9
	strong taste ***	42	39	47	59	9	17	18	55
Flavor	milk flavor ***	10	8	7	1	32	12	38	7
	vanilla flavor ***	4	7	4	2	29	5	15	4
Texture	thin ***	18	50	33	24	31	33	44	37
	thick ***	38	4	27	24	17	20	14	13
	floury ***	25	2	18	14	12	10	14	10

^n.s.^, non-significant difference according to Cochran’s Q test. significant difference for * *p* < 0.05; *** *p* < 0.001.
